# CBCT‐Assisted Clinic and Radiographic Diagnosis of an Atypically Erupted Odontoma Associated With Supernumerary Tooth

**DOI:** 10.1155/crid/3090872

**Published:** 2026-03-06

**Authors:** Marina Nunes de Faria Corrêa, Pedro Schwartz Kalil Pereira, Marcos Antonio Torriani, Josué Martos, Melissa Feres Damian

**Affiliations:** ^1^ Postgraduate Program in Health Sciences, Federal University of Rio Grande, Rio Grande, Rio Grande do Sul, Brazil, furg.br; ^2^ Postgraduate Program in Dentistry (Oral Diagnosis), School of Dentistry, Federal University of Pelotas, Pelotas, Rio Grande do Sul, Brazil, ufpel.edu.br; ^3^ Department of Oral and Maxillofacial Surgery and Traumatology, School of Dentistry, Federal University of Pelotas, Pelotas, Rio Grande do Sul, Brazil, ufpel.edu.br; ^4^ Department of Oral Medicine and Clinic, School of Dentistry, Federal University of Pelotas, Pelotas, Rio Grande do Sul, Brazil, ufpel.edu.br

**Keywords:** cone-beam computed tomography, odontoma tooth, supernumerary tooth

## Abstract

Odontomas are common benign odontogenic tumors and are typically asymptomatic, often detected incidentally through imaging exams. However, in some instances, these lesions may erupt into the oral cavity and coexist with supernumerary teeth, creating diagnostic and therapeutic challenges and interfering with normal tooth eruption. This case reports an unusual and complex presentation of an odontogenic lesion with clinic and radiographic features consistent with a partially erupted compound odontoma, associated with a supernumerary tooth and an embedded permanent lateral incisor. A 28‐year‐old female was referred to a dental school for evaluation of a recurrent, asymptomatic whitish spicule in the anterior mandibular gingiva. Initial radiographic examination revealed multiple radiopaque, denticle‐like structures, in addition to a vertically oriented supernumerary tooth and an embedded left mandibular lateral incisor. Based on a two‐dimensional imaging, a peripheral compound odontoma was initially suspected. Subsequent cone‐beam computed tomography (CBCT) scan provided critical three‐dimensional information, refining the imaging diagnosis and demonstrating an intraosseous compound odontoma with partial eruption into the oral cavity. Surgical removal of the embedded tooth was performed, while the supernumerary tooth and the odontogenic lesion, due to their lingual positioning and proximity to adjacent structures, were preserved. Later, the tooth erupted spontaneously, requiring a second intervention. Some residual denticles remained as a result of anatomical limitations. This case highlights the clinical, anatomical, and surgical complexity of erupted compound odontomas, underscores the indispensable role of CBCT in accurate radiographic diagnosis and treatment planning, and draws attention to potential challenges such as capsule rupture and incomplete removal of denticle‐like structures remnants.

## 1. Introduction

Odontomas are common benign odontogenic lesions, arising from fully differentiated odontogenic epithelial and mesenchymal tissues [1], and are currently regarded as hamartomas due to their slow and limited growth [2]. According to the histological and morphological organizations, odontomas are conventionally classified as compound, presenting multiple denticle‐like structures, or complex, characterized by an amorphous mass of dental tissue [3, 4]. Based on anatomical location, they may also be categorized as central (intraosseous) or peripheral (extraosseous) odontoma [5, 6]. Traumatic lesions, cell rests of Serres, factors and mutations genetics are accepted as possible etiological factors of the odontomas [2].

Epidemiological studies indicate that odontomas account for approximately 20%–67% of all odontogenic tumors, with higher prevalence during the second and third decades of life [7–9]. While some studies report no evidence of sexual dimorphism in the expression of this anomaly, others have observed a higher prevalence among females [10]. Compound odontomas are generally more frequent than complex odontomas and show a marked predilection for the anterior maxilla, whereas complex odontomas are more commonly found in the posterior mandible [3, 5, 7, 8, 11, 12]. In most cases, odontomas are asymptomatic and detected incidentally on routine radiographic examinations, often in association with delayed eruption or impaction of permanent teeth [13, 14].

Eruption of odontomas into the oral cavity is considered an exceptional clinical event and a limited number of cases have been reported in the English‐language literature [3, 5, 10, 11]. Unlike teeth, odontomas lack a periodontal ligament, and their eruptive behavior does not follow the physiological mechanism of tooth eruption [15–18]. The most frequent site of eruption for compound odontomas is the anterior maxilla, whereas complex odontomas tend to erupt in the posterior mandible [3, 6, 12, 15, 18].

Supernumerary teeth are another frequent developmental anomaly associated with eruption disturbances and are commonly found in anterior regions of the jaws [19]. Evidence suggests a potential overlap in the developmental pathways of odontomas and supernumerary teeth, with both linked to alterations in WNT/β‐catenin signaling [20, 21]. Moreover, some authors propose that compound odontomas may represent highly differentiated supernumerary teeth, particularly when composed of multiple denticles [10, 22]. The coexistence of these anomalies within the same region further complicates diagnosis and treatment [23].

Considering these findings, the present study reports an unusual case of a partially erupted intraosseous odontogenic lesion with clinic and radiographic features consistent with a compound odontoma in the anterior mandible, which revealed the presence of embedded supernumerary tooth and permanent mandibular left lateral incisor at the same region. This report emphasizes the impact of cone‐beam computed tomography (CBCT) on the radiographic diagnostic process of the case, initially suspected as a peripheral compound odontoma based on limited two‐dimensional radiographic examination.

## 2. Case Report

This case report was prepared in accordance with the CARE guidelines [24] (see Supporting Information). Following the recommendations of the International Committee of Medical Journal Editors (ICMJE), written informed consent was obtained from the patient, consenting to the publication of the case.

**Figure 1:**
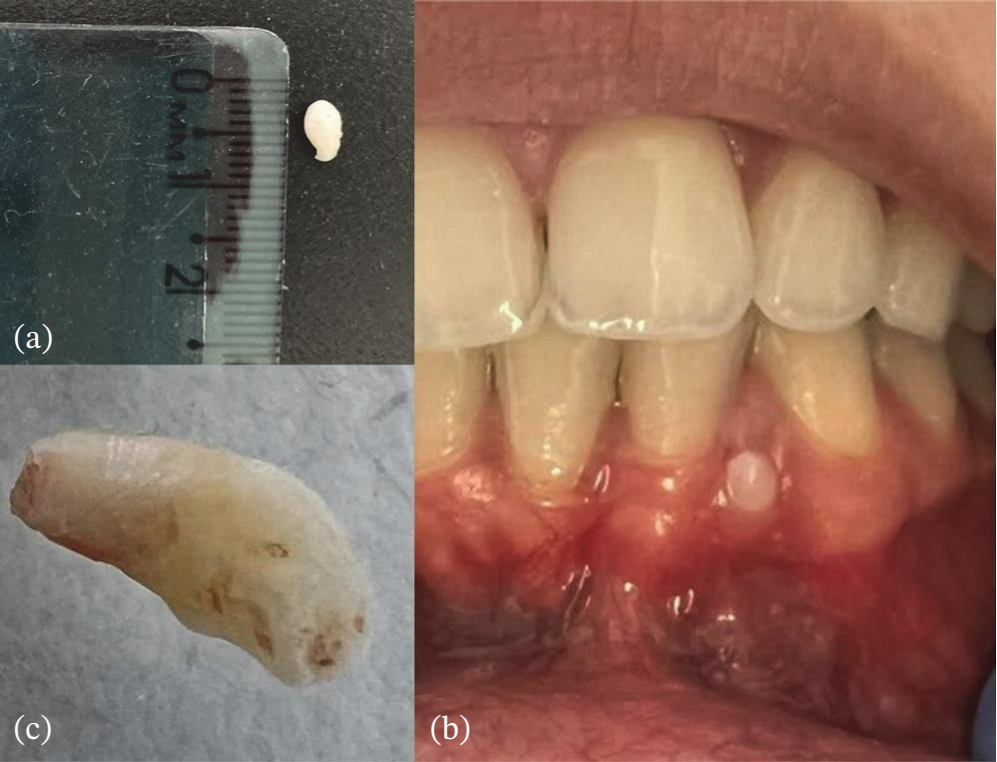
Initial clinical presentation. (a) First structure removed from the anterior lingual mandibular region. (b) Second structure in the anterior buccal region, resembling a rudimentary tooth crown. (c) Dentiform aspect of the second removed structure.

A 28‐year‐old female patient initially presented to a private dental clinic in the second half of 2022 with a small, asymptomatic, whitish spicule located in the lingual gingival region of the mandibular anterior teeth. The patient reported no systemic impairment or other relevant medical condition, and her history was unremarkable except for orthodontic treatment performed during adolescence. Based on its clinical appearance and the concomitant presence of mineralized biofilm in other areas of the oral cavity, the lesion was clinically interpreted as dental calculus and removed during supragingival scaling, without the need for complementary imaging examinations (Figure 1a). In November 2022, a second lesion with similar characteristics appeared on the buccal aspect of the same region (Figure 1b). Its removal, performed by the same private practitioner, revealed a hard structure resembling a small rudimentary tooth (Figure 1c). Given the recurrence and the atypical morphology of the removed structures, the patient was referred in July 2023 to a dental school for further diagnostic investigation.

At the academic institution, clinical examination revealed the absence of the left mandibular lateral incisor, with no history of extraction (Figure 2a). Periapical and occlusal radiographs were then obtained, demonstrating multiple irregular radiopaque structures in the anterior mandibular alveolar ridge, between the left central incisor and cuspid, in association with a vertically positioned supernumerary tooth and an embedded left mandibular lateral incisor (Figure 2b,c). Therefore, a clinic and radiographic diagnosis of a lesion with features consistent with a peripheral compound odontoma associated with a supernumerary tooth was initially established. Both alterations were considered responsible for the prolonged retention of the mandibular lateral incisor.

Figure 2Baseline clinical and radiographic assessment at the dental school. (a) Initial clinical aspect of the anterior mandibular region, showing the absence of the lateral incisor. (b) Periapical and (c) occlusal radiographs demonstrating an embedded mandibular left lateral incisor associated with a vertically submucosal supernumerary tooth, as well as multiple dense structures exhibiting radiographic features consistent with dental tissues of a peripheral compound odontoma.

**Figure:**
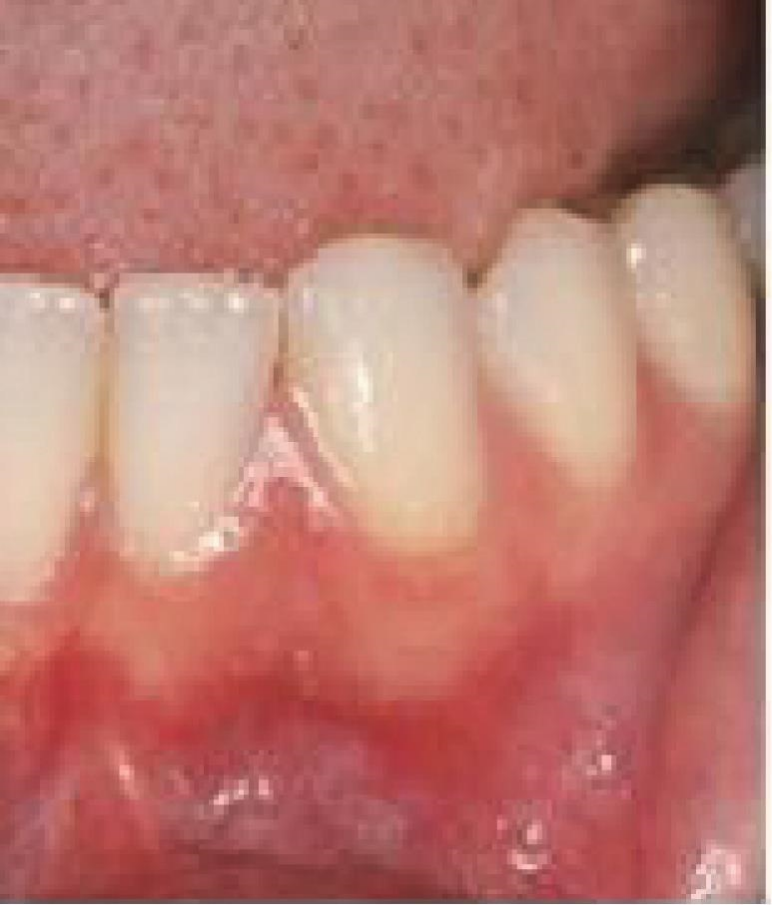
(a)

**Figure:**
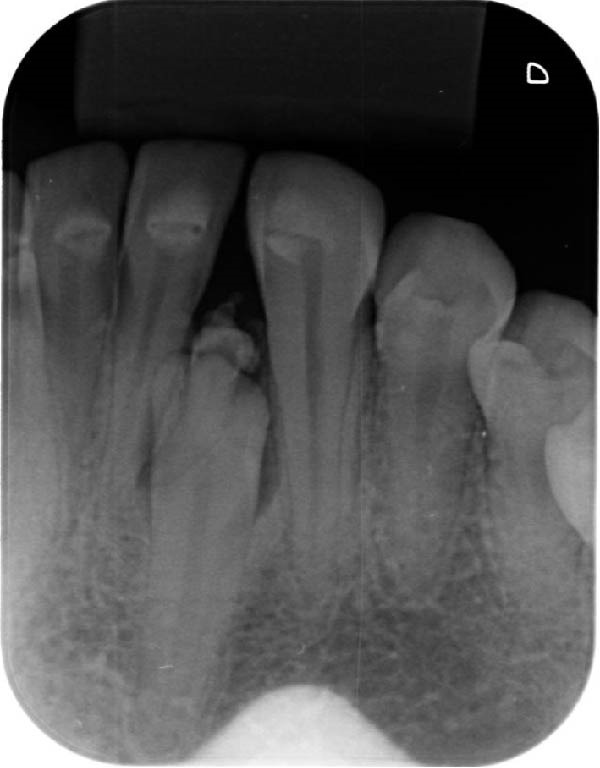
(b)

**Figure:**
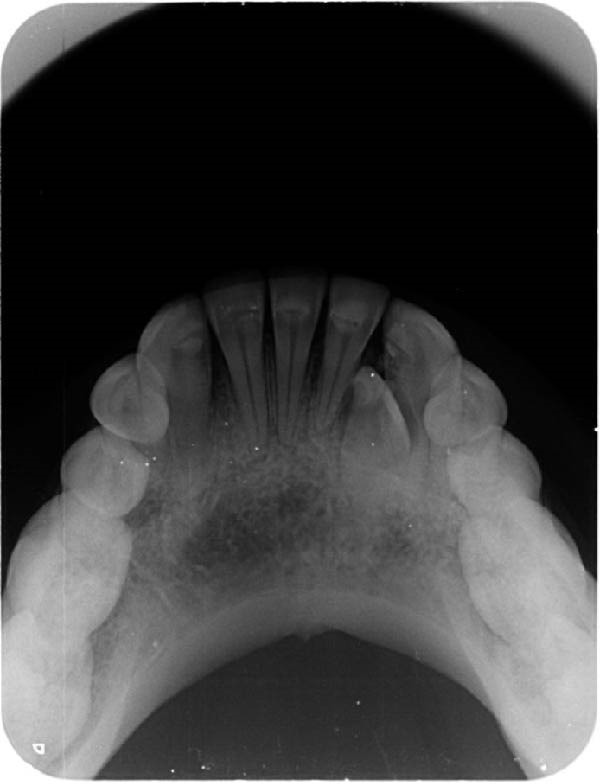
(c)

**Figure 3:**
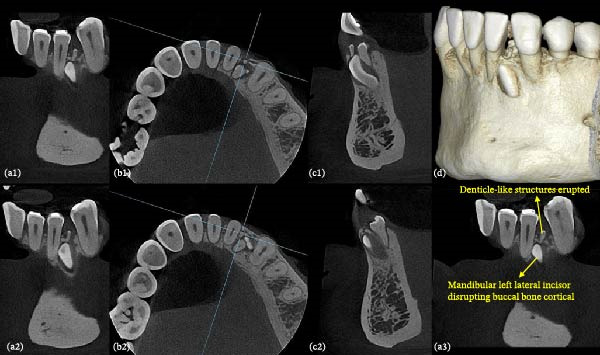
CBCT assessment. (a1, a2, a3) Coronal, (b1, b2) axial, and (c1, c2) sagittal planes, as well as (d) a three‐dimensional reconstruction, showing the spatial relation between the embedded lateral incisor, the supernumerary tooth, and the denticle‐like structures compatible with a compound odontoma. Cortical bone disruption at the alveolar ridge supports the suspicion of a partially erupted intraosseous compound odontoma.

**Figure 4:**
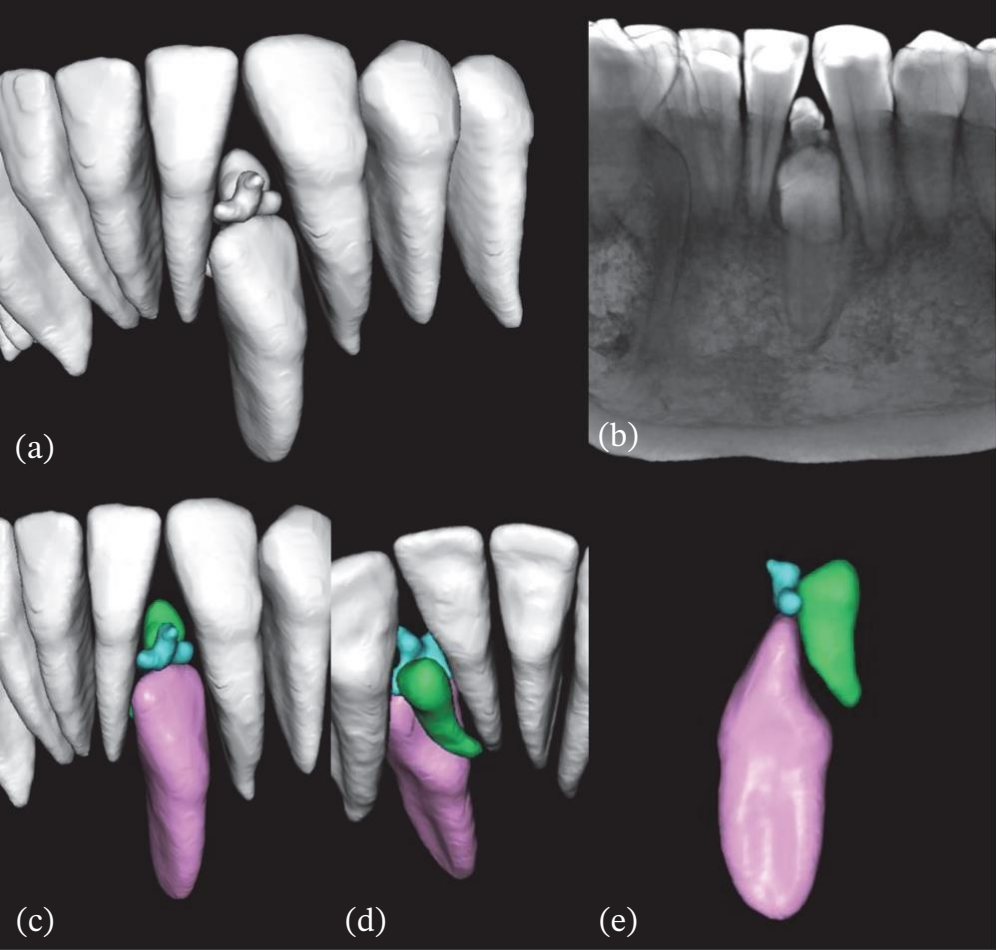
Three‐dimensional reconstruction and segmentation of the denticle‐like structures compatible with the compound odontoma, supernumerary teeth, and embedded lateral incisor in the anterior mandible. (a) Three‐dimensional segmentation of the involved structures. (b) Three‐dimensional reconstruction of the anterior mandibular region. (c) Coronal view highlighting the spatial relationship between the odontoma/supernumerary teeth and the lateral incisor. (d) Lingual view showing the impaction pathway and morphological details. (e) Isolated segmentation depicting the complete morphology of all segmented structures.

To further characterize the lesion and support treatment planning, a CBCT scan with a limited FOV was performed in July 2023 (Figures 3 and 4), as conventional two‐dimensional radiographs were insufficient to accurately determine the position and extent of the lesion, as well as its relationship to both permanent and supernumerary teeth. The images revealed a lingually positioned supernumerary tooth in association with multiple hyperdense structures exhibiting radiographic features consistent with denticles of a compound odontoma. Some structures were extraosseous, while others remained intraosseous, with cortical disruption, particularly on the buccal surface and alveolar ridge. These three‐dimensional imaging findings led to a revision of the initial radiographic diagnostic hypothesis, indicating that the lesion was in fact an intraosseous compound odontoma that had partially erupted into the oral cavity.

**Figure 5:**
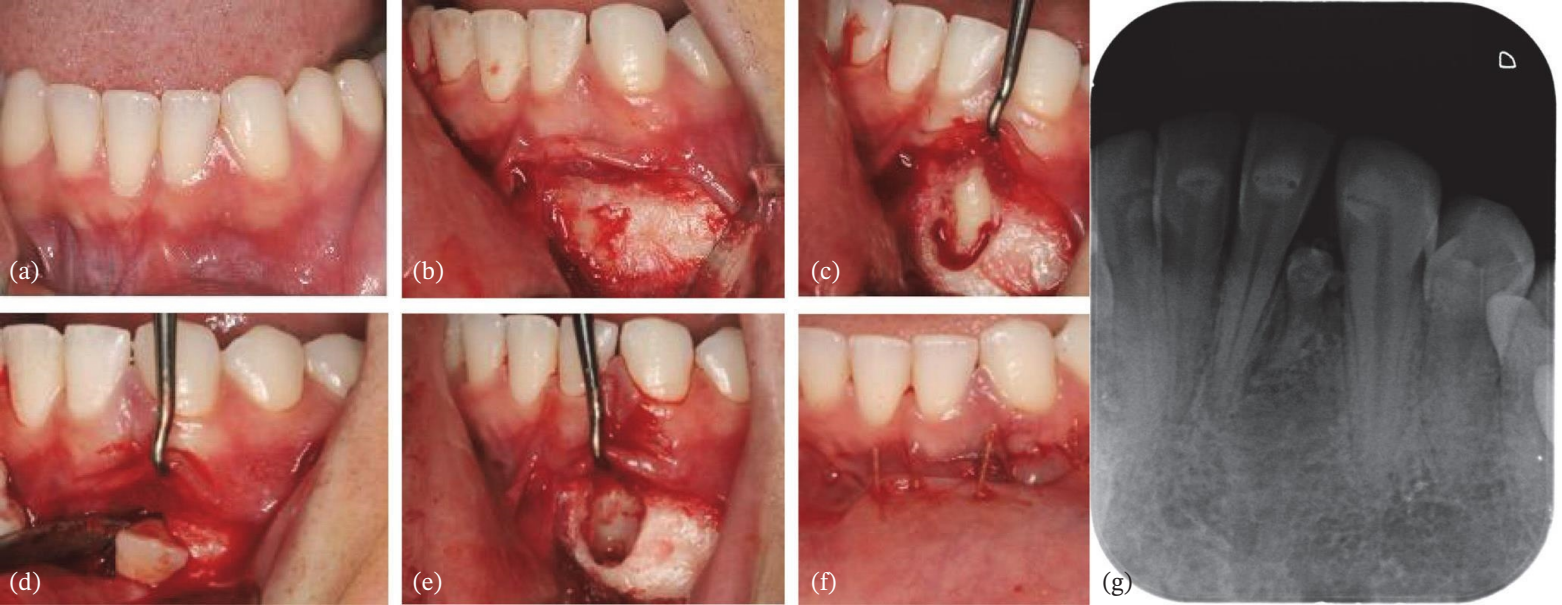
Surgical intervention and follow‐up. (a–f) Removal of the embedded lateral incisor via buccal access; limited access required a second lingual approach. (g) One‐year follow‐up radiograph showing persistence of the supernumerary tooth and residual denticle‐like structures.

Surgical removal of the odontoma and the supernumerary tooth was indicated. Considering the patient’s previous orthodontic treatment and minor esthetic concern, the extraction of embedded lateral incisor was also planned, with patient consent. The procedure was performed in August 2023 through a vestibular approach (Figure 5a–f), given the buccal position of permanent lateral incisor, observed on the CBCT scan. Due to the proximity of the supernumerary tooth to adjacent teeth and the need for a new lingual access, in view of its lingual position, the supernumerary was preserved to avoid iatrogenic damage. As a consequence of this conservative surgical approach, combined with the poor degree of mineralization of the denticle‐like structures and their close relationship with adjacent permanent teeth, no tissue suitable for curettage or histopathological examination could be obtained. The patient was informed about the persistence of the supernumerary and the potential need for a future surgical intervention for its removal.

At follow‐up in September 2024, the region remained asymptomatic and periodontally stable. A periapical radiograph (Figure 5g) confirmed the persistence of the supernumerary tooth in a submucosal position, as well as circular radiopaque images of varying densities located above the crown of the supernumerary tooth, between central incisor and cuspid, corresponding to the residual denticles of compound odontoma previously diagnosed on radiographic ground, that was not removed. The patient agreed to a second surgical procedure but chose to postpone it for professional reasons.

**Figure 6:**
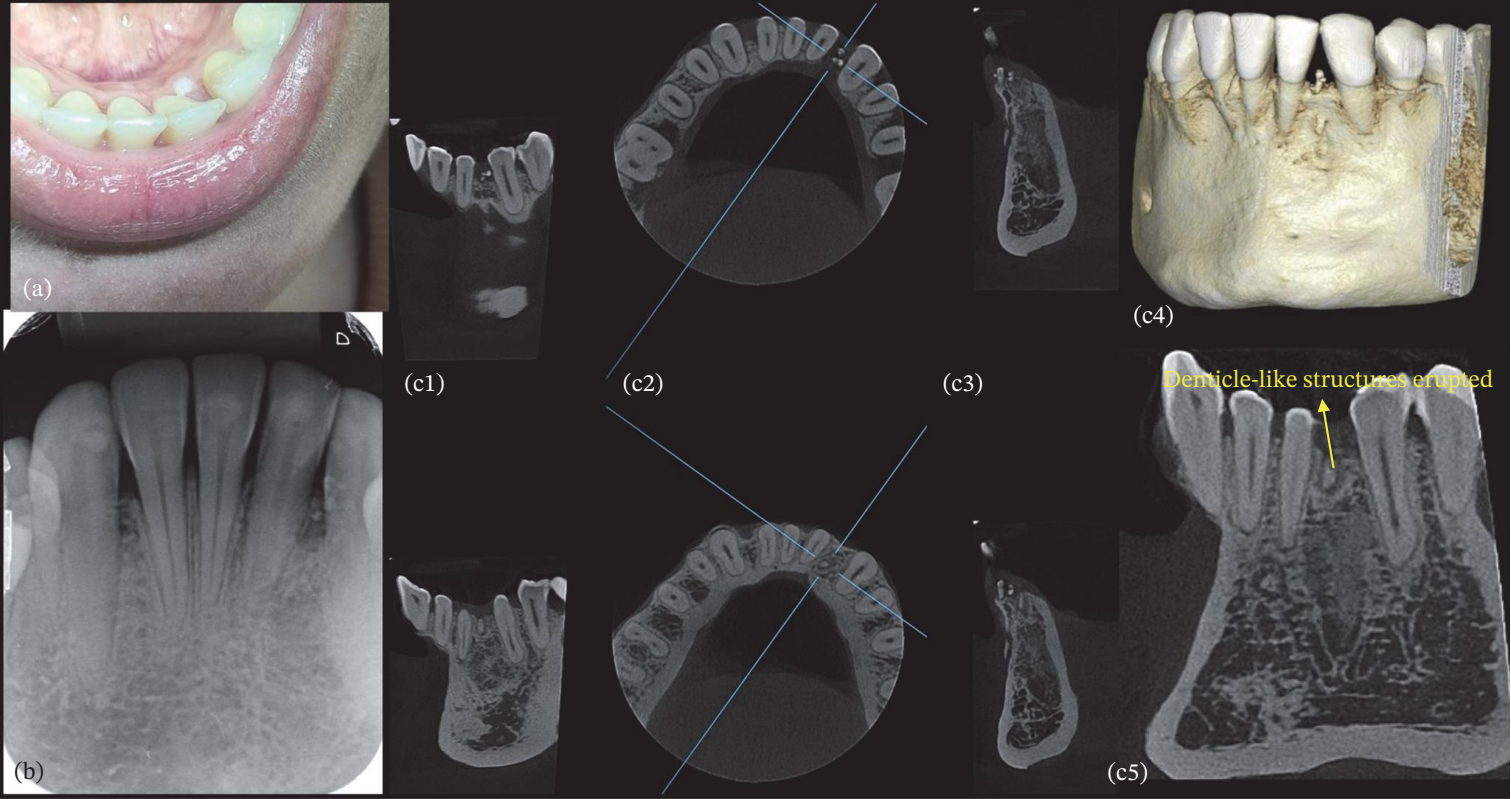
Clinical and tomographic follow‐up. (a) Eruption of the supernumerary tooth in the lingual region. (b) Postoperative radiographic follow‐up showing residual denticle‐like structures. (c) Coronal (c1, c5), axial (c2), and sagittal (c3) planes, as well as three‐dimensional reconstruction (c4), CBCT follow‐up, demonstrating the presence of residual denticle‐like structures and their proximity with adjacent permanent teeth.

In February 2025, the patient returned, reporting eruption of the supernumerary tooth in the lower lingual region (Figure 6a), which was extracted without complications, in March 2025. A follow‐up radiograph, performed in March 2025 (Figure 6b) confirmed its complete removal but revealed persistent radiopaque images consistent with residual component of the lesion. A new CBCT scan performed in May 2025 (Figure 6c) revealed hyperdense areas corresponding to small denticle‐like structures with radiographic features of a compound odontoma, some of which remained intraosseous, while others were located submucosally and in close proximity to the permanent teeth (left mandibular central incisor and cuspid). Due to the difficulty of removing these very small and poorly mineralized structures, and in agreement with the patient, no further surgical intervention was undertaken. The patient remains under clinical follow‐up owing to the possibility of spontaneous eruption of the residual denticles exhibiting clinic and radiographic features consistent with the odontogenic lesion.

## 3. Discussion

Although erupted odontomas have been previously reported, this clinical presentation remains rare and is consistently described as unusual, even within case reports [3, 10, 12, 14]. In the present case, the eruption was considered atypical not merely because of lesion exposure to the oral cavity, but due to the combined presence of partial eruption from an intraosseous origin, cortical bone disruption, simultaneous intraosseous and extraosseous components, and the association with both a supernumerary tooth and an embedded permanent lateral incisor in the same anatomical region. Taken together, these features, particularly as revealed by CBCT, underscore the diagnostic and clinical singularity of this case and contribute meaningful evidence to the limited literature on erupted odontomas.

When compared with previously reported cases of erupted odontomas, the present case shares some clinical characteristics described in the literature, such as the absence of painful symptoms; however, the findings were not incidental, as exposure of the lesion to the oral cavity prompted the patient to seek dental care. From an epidemiological perspective, previous studies indicate that erupted odontomas predominantly affect young individuals, most frequently during the first two decades of life, and exhibit a slight female predilection, which is consistent with the profile of the patient in the present report. However, in contrast to the pattern most commonly described in the literature, where compound odontomas preferentially erupt in the anterior maxilla, the odontogenic lesion reported herein erupted in the anterior mandible, a location considered less frequent [3, 6, 11, 12, 18].

The mechanism of eruption of the odontomas into the oral cavity remains poorly elucidated, given the absence of a periodontal ligament and deviation from the physiological eruption pathway. Proposed mechanisms, including progressive bone resorption, alveolar remodeling, capsular expansion, and cortical bone thinning, as well as eruptive forces from adjacent impacted teeth [3, 6, 12, 17, 18]. In the present case, the concurrent presence of a supernumerary tooth and an embedded lateral incisor may have generated localized pressure, facilitated the bone resorption and contributed to the partial eruption of the clinic and radiographically diagnosed compound odontoma.

In addition to the partial eruption of the clinic and radiographically diagnosed odontogenic lesion, this report describes the presence of a supernumerary tooth in the same anatomical region of the odontogenic lesion. This finding supports the growing body of evidence suggesting overlapping characteristics between compound odontomas and supernumerary teeth, not only in terms of morphology, clinical and histological aspects, but also in developmental origin. Some authors suggest that compound odontomas may represent the terminal stage of a developmental continuum involving supernumerary teeth, in which a disorganized, highly differentiated dental structure manifests as either a malformed supernumerary tooth or a compound odontoma [10, 22, 25]. The coexistence of both anomalies in the same anatomical site, as observed in this case, reinforces this hypothesis and underscores the need for thorough imaging and clinical evaluation. Moreover, it raises considerations about shared molecular pathways, such as WNT/β‐catenin, BMP and FGF signaling, potentially involved in their development [20, 21].

Clinically, most cases of odontomas reported in the literature are diagnosed incidentally through imaging exams [3, 13]. In contrast, in the present case, it was the partial eruption of the lesion itself that prompted clinical investigation and ultimately led to the diagnosis. Despite this atypical presentation, imaging played a fundamental role in confirming the presence of the odontogenic anomaly and in identifying its full extent in this case. Radiographic and tomographic examinations not only revealed the odontoma but also enabled the detection of a supernumerary tooth and a retained permanent lateral incisor.

Still discussing the importance of imaging exams in this report, the CBCT scan proved to be a valuable tool for accurate radiographic characterization of the case, as previously cited in the literature [10]. While conventional two‐dimensional radiographs remain useful for the initial detection of odontogenic lesion, they are inherently limited by image superimposition and by the inability to depict buccolingual relationship and cortical bone integrity. In the present case, these limitations initially suggested the presence of a peripheral (extraosseous) compound odontoma. In contrast, CBCT provided decisive diagnostic information by enabling multiplanar evaluation which clearly demonstrated the intraosseous origin of the lesion, its partial eruption through cortical bone disruption, and its close spatial relationship with adjacent permanent teeth and the supernumerary tooth, as well as the supernumerary tooth with the embedded lateral incisor. This information was critical not only for refining the radiographic diagnosis, but also directly influencing surgical planning and supporting a conservative management strategy.

Surgical enucleation remains the treatment of choice for odontomas, specially to adult patients, and is widely acknowledged for its favorable clinical outcomes [2, 18]. However, in this case the intervention revealed surgical challenges, particularly the incomplete removal of the odontoma, likely due to rupture of its fibrous capsule during eruption. This may have led to the intraoperative dispersion of denticles, increasing the difficulty of enucleation and the likelihood of residual fragments, a complication previously reported in cases of erupted odontomas, which often lose their defined cortical boundaries and present with less predictable surgical behavior [18].

A limitation of this study was the absence of histopathological confirmation, as odontoma are classically defined as histopathological entities. In the present case, however, histological analysis could not be performed due to the proximity of the denticle‐like structures to the adjacent permanent teeth (central incisor and cuspid), their poor degree of mineralization and their scattered distribution, as well as the rupture of the lesion capsule during the eruptive process. To prevent iatrogenic damage, only the dental structures (the permanent lateral incisor and the supernumerary tooth) were removed during the surgical procedures, and no additional tissue suitable for curettage or microscope evaluation was available. Despite this limitation, the diagnosis was established on robust clinic and radiographic grounds, due to the CBCT examination. Compound odontomas exhibit highly characteristic imaging features, particularly when assessed using CBCT, including multiple well‐organized denticles with radiodensity comparable to enamel and dentin, frequently surrounded by a narrow radiolucent rim corresponding to the fibrous capsule [3, 8, 21, 22]. These pathognomonic features were clearly observed in the present case. Moreover, that compound odontomas are composed of normal dental tissues, their histological differentiation from supernumerary teeth may be challenging, even when microscopic analysis is available [22]. Similar diagnostic approaches based on consistent clinical and imaging findings, in the absence of histopathological examination, have been reported in the literature [2, 10–12, 18].

In conclusion, this clinical case highlights the diagnostic, anatomical, and therapeutic complexity associated with compound odontomas, particularly when partially erupted into the oral cavity and accompanied by supernumerary and embedded permanent teeth in the same region. Although unusual, the eruption of odontomas should be considered in the differential diagnosis of hard‐tissue lesions emerging through the mucosa. The case also reinforces the value of CBCT as a decisive tool for radiographic diagnosis, accurately assessing spatial relationships, and guiding surgical planning.

## Funding

No funding was received for this study.

## Ethics Statement

Following the recommendations of the International Committee of Medical Journal Editors (ICMJE), written informed consent was obtained from the patient, consenting to the publication of the case.

## Conflicts of Interest

The authors declare no conflicts of interest.

## Supporting Information

Additional supporting information can be found online in the Supporting Information section.

## Supporting information


**Supporting Information** Completed CARE checklist demonstrating compliance with the CARE guidelines for case report reporting.

## Data Availability

Data sharing is not applicable to this article as no datasets were generated or analyzed during the current study.

## References

[1] Vered M. and Wright J. M. , Update From the 5th Edition of the World Health Organization Classification of Head and Neck Tumors: Odontogenic and Maxillofacial Bone Tumours, Head and Neck Pathology. (2022) 16, no. 1, 63–75, 10.1007/s12105-021-01404-7. 35312978 PMC9019005

[2] Apablaza J. A. , Munõz G. , Arriagada C. , Bucchi C. , Masuko T. , and Fuentes R. , Odontoma Recurrence. The Importance of Radiographic Controls: Case Report With a 7-Year Follow-Up, Medicina. (2024) 60, no. 8, 10.3390/medicina60081248, 1248. 39202528 PMC11356190

[3] Bereket C. , Çakir-Özkan N. , Şener I. , Bulut E. , and Tek M. , Complex and Compound Odontomas: Analysis of 69 Cases and a Rare Case of Erupted Compound Odontoma, Nigerian Journal of Clinical Practice. (2015) 18, no. 6, 726–730, 10.4103/1119-3077.154209, 2-s2.0-84940553562. 26289508

[4] Soluk-Tekkesin M. , Bologna-Molina R. , and Magliocca K. , et al.Malformations vs. Neoplasia in the Oral Cavity: Special Emphasis on Mixed Odontogenic Tumors, Journal of Oral Pathology & Medicine. (2025) 54, no. 1, 76–79, 10.1111/jop.13592. 39617625

[5] Junquera L. , de Vicente J. C. , Roig P. , Olay S. , and Rodríguez-Recio O. , Intraosseous Odontoma Erupted Into the Oral Cavity: An Unusual Pathology, Medicina oral, patologia oral y cirugia bucal. (2005) 10, no. 3, 248–251. 15876969

[6] Honnegowda D. K. K. , Ranganna V. L. , Chandregowda K. Y. , Krishna G. , and Kumar N. , Erupted Compound Odontoma: A Rare Case Report and Review, International Journal of Oral Care and Research. (2019) 7, no. 3, 77–80, 10.4103/INJO.INJO_34_19.

[7] Sanchéz O. H. , Berrocal M. I. L. , and González J. M. M. , Metanalysis of the Epidemiology and Clinical Manifestations of Odontomas, Medicina Oral Patologia Oral Y Cirugia Bucal. (2008) 13, no. 11, E730–E734. 18978716

[8] Kämmerer P. W. , Schneider D. , and Schiegnitz E. , et al.Clinical Parameter of Odontoma With Special Emphasis on Treatment of Impacted Teeth – A Retrospective Multicentre Study and Literature Review, Clinical Oral Investigations. (2016) 20, no. 7, 1827–1835, 10.1007/s00784-015-1673-3, 2-s2.0-84948437175. 26612404

[9] Borghesi A. , Tonni I. , Pezzotti S. , and Maroldi R. , Peripheral Osteoma, Compound Odontoma, Focal Cemento-Osseous Dysplasia, and Cemento-Ossifying Fibroma in the Same Hemimandible: CBCT Findings of an Unusual Case, Radiology Case Reports. (2017) 12, no. 4, 756–759, 10.1016/j.radcr.2017.08.011, 2-s2.0-85029600554. 29484064 PMC5823294

[10] Yordanova-Kostova G. R. and Gurgurova G. D. , Compound Odontoma in Canine Region and Its Radiological Evidence: Case Reports, The Open Dentistry Journal. (2023) 17, no. 1, 10.2174/18742106-v17-e230911-2023-84.

[11] Serra-Serra G. , Berini-Aytés L. , and Gay-Escoda C. , Erupted Odontomas, a Report of Three Cases and Review of the Literature, Medicina Oral Patologia Oral Y Cirugia Bucal. (2009) 14, no. 6, E299–E303. 19300370

[12] Agarwal S. , Rao S. , Lepcha J. , and Galhotra V. , Large Erupted Complex Odontoma Mimicking Maxillary Osteomyelitis, BMJ Case Reports. (2023) 16, no. 1, 10.1136/bcr-2022-253322. PMC9872485 36669786

[13] Isola G. , Cicciù M. , Fiorillo L. , and Matarese G. , Association Between Odontoma and Impacted Teeth, Journal of Craniofacial Surgery. (2017) 28, no. 3, 755–758, 10.1097/SCS.0000000000003433, 2-s2.0-85010004864. 28468159

[14] Marya A. and Venugopal A. , Impaction Caused by a Rare Erupted Peripheral Compound Odontoma, Clinical Case Reports. (2021) 9, no. 11, 10.1002/ccr3.5158. PMC8617333 34853693

[15] Vengal M. , Arora H. , Ghosh S. , and Pai K. M. , Large Erupting Complex Odontoma: A Case Report, Journal of the Canadian Dental Association. (2007) 73, no. 2, 169–173. 17355809

[16] Shekar S. E. , Rao R. S. , Gunasheela B. , and Supriya N. , Erupted Compound Odontome, Journal of Oral and Maxillofacial Pathology. (2009) 13, no. 1, 47–50, 10.4103/0973-029X.48758. 21886999 PMC3162857

[17] Hanemann J. A. C. , Oliveira D. T. , Garcia N. G. , Santos M. R. G. , and Pereira A. A. C. , Peripheral Compound Odontoma Erupting in the Gingiva, Head & Face Medicine. (2013) 9, no. 1, 9–15, 10.1186/1746-160X-9-15, 2-s2.0-84878708594. 23758697 PMC3684544

[18] Sarojini S. B. , Khosla E. , Varghese T. , and Johnson Arakkal L. , Eruption of Odontomas Into the Oral Cavity: A Report of 2 Cases, Case Reports in Dentistry. (2014) 2014, no. 1, 639173. 24900927 10.1155/2014/639173 PMC4037568

[19] Jang D. H. , Chae Y. K. , and Lee K. E. , et al.Determination of the Range of Intervention Timing for Supernumerary Teeth Using the Korean Health Insurance Review and Assessment Service Database, Journal of Clinical Pediatric Dentistry. (2022) 47, no. 1, 67–73, 10.22514/jocpd.2022.036. 36627222

[20] Lu X. , Yu F. , and Liu J. , et al.The Epidemiology of Supernumerary Teeth and the Associated Molecular Mechanism, Organogenesis. (2017) 13, no. 3, 71–82, 10.1080/15476278.2017.1332554, 2-s2.0-85045952115. 28598258 PMC5654855

[21] Liu S. , Lin Z. , Wen S. , Teng Y. , Xie K. , and Huang Y. , Epidemiological and CBCT Characterizations of Odontomas: A Retrospective Study of 87,590 Subjects, Oral Diseases. (2024) 30, no. 7, 4585–4597, 10.1111/odi.14845. 38129744

[22] Pippi R. , Odontomas and Supernumerary Teeth: Is There a Common Origin?, International Journal of Medical Sciences. (2014) 11, no. 12, 1282–1297, 10.7150/ijms.10501, 2-s2.0-84921669508. 25419174 PMC4239149

[23] Kobayashi T. Y. , Gurgel C. V. , Cota A. L. , Rios D. , Machado M. A. A. , and Oliveira T. M. , The Usefulness of Cone Beam Computed Tomography for Treatment of Complex Odontoma, European Archives of Paediatric Dentistry. (2013) 14, no. 3, 185–189, 10.1007/s40368-013-0036-5, 2-s2.0-84893005067. 23633234

[24] Gagnier J. J. , Kienle G. , Altman D. G. , Moher D. , Sox H. , and Riley D. , The CARE Guidelines: Consensus-Based Clinical Case Reporting Guideline Development, Journal of Medical Case Reports. (2013) 7, no. 1, 10.1186/1752-1947-7-223, 2-s2.0-84884315841, 223. 24228906 PMC3844611

[25] Elsayed L. K. , El Khateeb S. M. , Alzahrani S. A. , ALHarthi S. S. , and Ba-Hattab R. , Case Report: An Association of the Gubernacular Canal, Supernumerary Tooth and Odontoma With an Impacted Canine on Cone-Beam Computed Tomography, F1000Research. (2021) 9, 1204. 10.12688/f1000research.26627.1 PMC7863995 33604026

